# Work exposure and associated risk of hospitalisation with pneumonia and influenza: A nationwide study

**DOI:** 10.1177/1403494820964974

**Published:** 2020-10-30

**Authors:** Lauge Østergaard, Rikke Nørmark Mortensen, Kristian Kragholm, Michael Dalager-Pedersen, Kristoffer Koch, Lars Køber, Christian Torp-Pedersen, Emil Fosbøl

**Affiliations:** 1 The Heart Centre, Rigshospitalet, Denmark; 2 Department of Clinical Epidemiology and Department of Cardiology, University of Aalborg, Denmark; 3 Epidemiology and Biostatistics, Aalborg University Hospital, Denmark; 4 Department of Infectious Diseases, Aalborg University Hospital, Denmark

**Keywords:** Pneumonia, influenza, work exposure, population-based study

## Abstract

**Background:**

Pneumonia and influenza are major health concerns and constitute a high economic burden. However, few data are available on the associated risk of pneumonia and influenza and work exposure on a large population scale.

**Aim:**

This study aimed to examine the associated risk of pneumonia and influenza by type of work exposure.

**Methods:**

By cross-linking administrative Danish registries, we classified people in 10 different profession types. The main outcome was hospitalisation with pneumonia or influenza. A multivariable Poisson regression analysis was used to assess the associated incidence rate ratio (IRR) of being hospitalised with pneumonia or influenza by type of profession.

**Results:**

A total of 1,327,606 people added risk time to the analyses. In a multivariable model, work in day care, public transportation, sewers and nursing home care was associated with an increased risk of hospitalisation with pneumonia compared to work within public administration: IRR=1.20 (95% confidence interval (CI) 1.12–1.28), IRR=1.21 (95% CI 1.09–1.34), IRR=1.61 (95% CI 1.19–2.19) and IRR=1.10 (95% CI 1.03–1.18), respectively. In a multivariable analysis, people working within public transportation were associated with an increased risk of hospitalisation with influenza compared to people working within public administration: IRR=2.54 (95% CI 1.79–3.58).

**Conclusions:**

**Working in day care, public transportation, sewers and nursing home care increased the associated risk of hospitalisation with pneumonia, and working within public transportation increased the associated risk of being hospitalised with influenza compared to working within public administration.**

## Introduction

Pneumonia and influenza are major health concerns. Pneumonia has been estimated to cost €10.1 billion in Europe, and the cost of lost work days has been estimated to be €3.6 billion annually, while the cost of influenza has been estimated at US$19,800 million [1–3]. Preventive means are necessary in order to lower this health concern and economic burden. However, before preventive means can be constituted, the magnitude of the problem of pneumonia and influenza by type of profession needs to be further assessed. The length of the average working week in Europe is 40.3 hours, constituting a potential risk period for contracting respiratory tract infections [4]. Prior studies have identified an increased risk of influenza in manual workers, janitors and cleaners, and secretaries. However, no studies have been able to investigate this on a large scale [5,6]. Other studies have investigated the social contact patterns and related this to an increased risk of influenza [7,8]. It is known from prior studies that living in a household of more than 10 people and living with children increase the risk of community-acquired pneumonia [9]. However, little is known about the associated risk of pneumonia and influenza for professions with many person contacts and professions working with children. Such knowledge can help us in the guidance of preventive and prophylactic means. We hypothesised that professions which typically include working with children and with direct person-to-person contact would be associated with an increased rate of pneumonia and influenza, as case-control studies have identified these as factors associated with an increased risk of pneumonia and influenza [10–12]. We set forth to investigate the associated risk of being hospitalised with pneumonia or influenza by type of profession.

## Methods

### Data sources

In Denmark, every Danish citizen is provided with a unique identifier, which makes it possible to cross-link different nationwide registries [13]. Linkages between registries have no mismatches. In this study, we acquired data from six registries. The first registry was the Danish Welfare Registry, which is maintained by the Danish Ministry of Occupation. This registry was established in 1991, and since 2008, it has registered profession type on a monthly basis for every Danish citizen who previously received financial support from the government. The registry includes around five million people for the period from 1991 onwards, and the estimated number of inhabitants in Denmark was around 5.7 million in 2016 [14,15]. The Danish welfare system provides financial support for students, people on maternity leave, people without employment, sickness benefit for illness lasting more than two weeks, childcare benefit and so on. The second registry used in this study was the National Patient Registry, which holds information on all hospitalisations in Denmark based on the International Classification of Diseases (ICD) from discharge papers. The registry covers inpatient visits since 1977, and from 1995, outpatient visit have been registered. This registry was used to identify the primary outcome (influenza and pneumonia) and co-morbidities of interest (see Supplemental Table SI for codes used), and has been described in detail previously [16]. The third registry used was the Cause of Death Registry, providing information on the date of death. The fourth registry was the Civil Registration System [13], and the fifth was the Danish Prescription Registry, which was used to define diabetes mellitus as any prescription filled on glucose-lowering medication six months prior to index (see Supplemental Table SI for codes used) [17]. Finally, the Register of Preventive Measures was used to assess if people were considered as living alone [18]. This register has been updated at the beginning of every new calendar year since 1986. The Register of Preventive Measures is based on two main parts, describing (a) socio-economic data on the Danish population and (b) health-care services not provided from hospitals.

### Study population

The study period was from 1 January 2008 to 31 December 2016. We used the Danish Population Registry to identify the study population of people who were 25–60 years of age at the start of the study period.

### Exposure

According to categorisation of profession types from the Danish Ministry of Occupation [19], we identified five professions with a low level of person-to-person contact and no work with children: the metal industry, farming and gardening, sewers, public administration and garbage and recycling. We also identified five professions with a higher rate of person-to-person contact, as well as professions that include working with children: day care, health-care workers, nursing home care, public schools and public transportation. Health-care workers were defined as people working in hospitals, general practitioners, consultants with private practice, physiotherapists and dentists.

### Outcome

The primary outcome of this study was hospitalisation with pneumonia or influenza assessed through the Danish National Patient Registry, which includes information on diagnosis codes according to the 10th edition of the ICD since 1994. We included only in-hospital, primary or secondary diagnosis codes (J09–J18) in the primary analysis. ICD-10 codes J12–18 have been validated in a cohort of cancer patients in the National Patient Registry, with a positive predictive value of 93% [20].

### Covariates

Co-morbidities (myocardial infarction, cancer, renal disease, peripheral vascular disease, heart failure, chronic obstructive pulmonary disease, rheumatic disease and atrial fibrillation) were assessed from the National Patient Registry as an inpatient or outpatient visit with a primary or secondary diagnosis. Glucose-lowering medication was assessed from the Prescription Registry. Sex and age were assessed from the Population Registry.

### Statistics

Study subjects were followed from the start of the study (1 January 2008) or the date of their 25th birthday until death, emigration, first date of hospitalisation with pneumonia or influenza or end of the study period, whichever came first. Only the first hospitalisation of pneumonia or influenza was assessed, and follow-up time was ended at the date of first admission. Risk time in each type of profession was computed for the total study population. Further, risk time for every co-morbidity (chronic obstructive pulmonary disease, peripheral vascular disease, heart failure, atrial fibrillation, rheumatic disease, cancer, diabetes and renal disease), age group (five-year intervals), living alone and calendar year were determined. The incidence rate of hospitalisation with pneumonia and influenza was computed, with risk time as the nominator and the total number of cases for each profession with 95% confidence intervals (CI). In multivariable adjusted Poisson regression analysis, incidence rate ratios (IRR) were computed for the comparison of the incidence rates for the different types of professions with ‘public administration’ as reference. Interaction with age on the primary outcome was tested using the maximum likelihood test. Two sensitivity analyses were conducted. In the first, only primary diagnosis of pneumonia and influenza was considered the main outcome in order to identify any differences within hospital coding. In the second, we included only patients >45 years of age. This analysis was conducted in order to identify differences within age groups. Statistical analyses were performed using SAS v9.4 (SAS Institute, Inc., Cary, NC) and RStudio [21].

### Ethics

Register-based studies do not need ethical approval in Denmark. The study was approved by the Danish Data Protection Agency.

## Results

The study cohort included 1,327,606 people who at some point during the study period were within one or more of the professions of interest. Among the included type of professions, public administration was the profession type with the most person years (923,745 PY), and sewers was the type of profession with the fewest person years (14,369 PY; Table I). Overall, the people included were healthy, with few co-morbidities. Differences were seen in the sex distribution between profession types, with the majority of females working in day care, public schools, public administration and nursing homes and as health-care workers at hospitals and dentists (Table I). For all profession types, ⩽2.0% of the observation time was for people with chronic obstructive pulmonary disease (Table I).

**Table I.** Baseline characteristics.

	Metal industry	%	Public administration	%	Public transport	%	Nursing home	%	Garbage and recycling	%	Day care	%	Public schools	%	Sewers	%	Farming and gardening	%	Health-care workers	%
Total PY	251,529		923,745		173,206		736,519		54,358		893,018		570,201		14,369		176,425		274,227	
Male, PY	207,774	82.6	271,899	29.4	145,157	83.8	133,777	18.2	45,580	83.9	172,053	19.3	174,125	30.5	11,775	81.9	127,274	72.1	36,434	13.3
Age 25–30, PY	20,094	8.0	72,673	7.9	9339	5.4	68,465	9.3	3284	6.0	89,465	10.0	41,223	7.2	903	6.3	45,669	25.9	24,584	9.0
Age 30–40, PY	62,186	24.7	217,951	23.6	31,619	18.3	158,577	21.5	11,120	20.5	221,985	24.9	152,783	26.8	2960	20.6	54,006	30.6	66,596	24.3
Age 40–50, PY	88,453	35.2	273,385	29.6	51,545	29.8	205,240	27.9	18,410	33.9	260,053	29.1	168,981	29.6	4957	34.5	40,525	23.0	81,020	29.5
Age 50–60, PY	66,767	26.5	278,897	30.2	60,139	34.7	244,065	33.1	17,275	31.8	258,764	29.0	157,002	27.5	4421	30.8	28,884	16.4	79,029	28.8
Age 60–70, PY	14,029	5.6	80,838	8.8	20,564	11.9	60,172	8.2	4270	7.9	62,750	7.0	50,212	8.8	1129	7.9	7341	4.2	22,998	8.4
AMI, PY	3022	1.2	9019	1.0	4459	2.6	7630	1.0	965	1.8	7722	0.9	5176	0.9	169	1.2	1365	0.8	1971	0.7
Cancer, PY	8536	3.4	45,471	4.9	7335	4.2	37,479	5.1	2220	4.1	43,239	4.8	28,388	5.0	557	3.9	4536	2.6	13,934	5.1
Renal disease, PY	1658	0.7	6223	0.7	1491	0.9	4810	0.7	423	0.8	5720	0.6	3612	0.6	147	1.0	830	0.5	1514	0.6
PVD, PY	2484	1.0	8822	1.0	2555	1.5	7667	1.0	709	1.3	8335	0.9	4973	0.9	128	0.9	1254	0.7	2268	0.8
Heart failure, PY	1644	0.7	6095	0.7	1916	1.1	4823	0.7	551	1.0	5566	0.6	3479	0.6	110	0.8	898	0.5	1377	0.5
COPD, PY	3143	1.2	11,898	1.3	3420	2.0	12,483	1.7	983	1.8	12,915	1.4	6861	1.2	206	1.4	1933	1.1	2969	1.1
Rheumatic disease, PY	3969	1.6	17,489	1.9	3944	2.3	14,583	2.0	1053	1.9	16,151	1.8	10,467	1.8	262	1.8	2179	1.2	5347	1.9
Diabetes, PY	10,858	4.3	44,118	4.8	15,297	8.8	38,750	5.3	3088	5.7	42,608	4.8	24,038	4.2	739	5.1	5059	2.9	10,655	3.9
Afib, PY	2900	1.2	10,883	1.2	2686	1.6	7856	1.1	892	1.6	9166	1.0	6544	1.1	165	1.1	1523	0.9	3080	1.1
Living alone, PY	66,741	26.5	221,542	24.0	55,918	32.3	215,365	29.2	15,434	28.4	257,063	28.8	130,710	22.9	3269	22.8	57,242	32.4	56,623	20.6

Patients were followed from study inclusion until: death, emigration, end of the study period (31 December 2016), hospitalisation with influenza or hospitalisation with pneumonia, whichever came first.

PY: person years; afib: atrial fibrillation; COPD: chronic obstructive pulmonary disease; AMI: acute myocardial infarction; PVD: peripheral vascular disease.

### Risk of pneumonia

**Figure 1.**
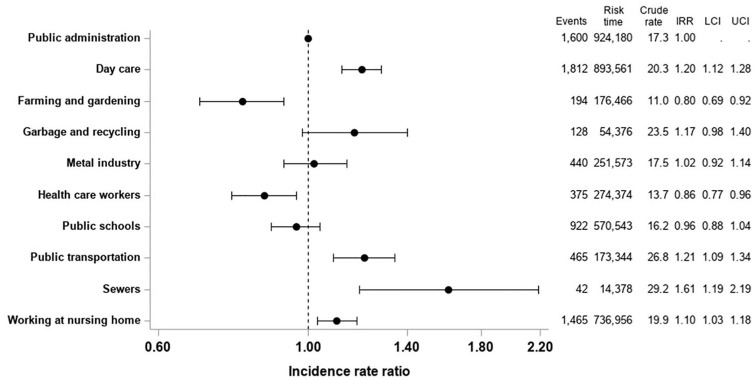
Risk of hospitalisation with pneumonia by profession type. Crude rate and adjusted incidence rate ratios of being hospitalized with pneumonia by type of profession.

Working within sewers, public transportation and garbage and recycling had the highest crude rate of being hospitalised with pneumonia: 29.2/10,000 PY, 26.8/10,000 PY and 23.5/10,000 PY, respectively (Figure 1). People working within farming and gardening, health-care workers at hospitals and dentists and those working at public schools had the lowest crude rate of being hospitalised with pneumonia: 11/10,000 PY, 13.7/10,000 PY and 16.2/10,000 PY, respectively (Figure 1). In adjusted analysis, we identified that people working in day care, sewers, public transportation and nursing home care had an increased rate of being hospitalised with pneumonia: IRR=1.20 (95% CI 1.12–1.28), IRR=1.61 (95% CI 1.19–2.19), IRR=1.21 (95% CI 1.09–1.34) and IRR=1.10 (95% CI 1.03–1.18) compared to people working within public administration (Figure 1).

### Risk of influenza

**Figure 2.**
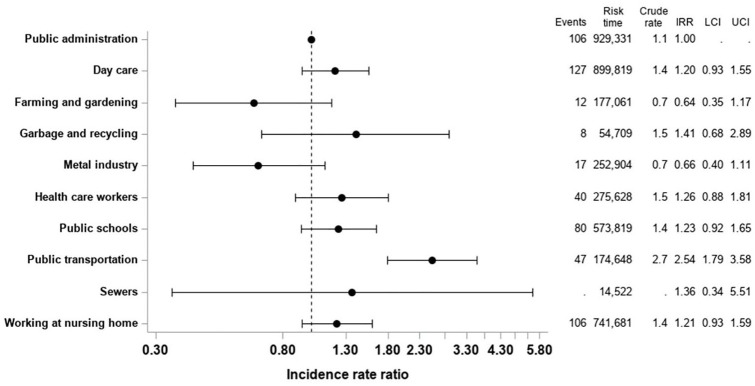
Risk of hospitalisation with influenza by profession type. Crude rate and adjusted incidence rate ratios of being hospitalized with influenza by type of profession.

The crude rate of being hospitalised with influenza was highest for people working within public transportation (2.7/10,000 PY) followed by people working within garbage and recycling (1.5/10,000 PY) and health-care workers at hospitals and dentists (1.5/10,000 PY; Figure 2). People working within farming and gardening and that metal industry had the lowest crude rate of being hospitalised with influenza (0.7/10,000 PY for both groups; Figure 2). In adjusted analysis, we identified that people working within public transportation were associated with an increased risk of being hospitalised with influenza compared to people working within public administration (IRR=2.54; 95% CI 1.79–3.58; Figure 2).

### Sensitivity analysis

For purposes of sensitivity, we only included hospitalisations with pneumonia or influenza categorised as a primary diagnosis. Overall, this did not change our main findings (Supplemental Figures S1 and S2). However, we identified that people working in public schools were at an increased risk of influenza (IRR=1.40; 95% CI 1.01–1.94) compared to people working within public administration. Further, we conducted a sensitivity analysis including only people ⩾45 years of age at study inclusion (1 January 2008). This decreased the crude rates. However, no changes were seen between profession types (Supplemental Figures S3 and S4).

## Discussion

We investigated the associated rate of hospitalisation with pneumonia and influenza by type of profession using Danish nationwide registries. Our study yielded two major findings. First, among 10 pre-specified professions, we identified that people working in day care, sewers, public transportation and nursing home care had an increased rate of hospitalisation with pneumonia compared to people working within public administration. When investigating hospitalisation with influenza, only people working within public transportation had an increased rate compared to people working within public administration.

Some studies have assessed the risk of pneumonia and influenza and type of work exposure. A case-control study from Spain included 1336 cases of community-acquired pneumonia [21]. The authors identified that more cases worked within construction compared to controls, whereas office workers had lower odds of community-acquired pneumonia. In a multivariable adjusted model, the authors identified no difference in type of profession. However, this study was limited by the case-control design with the risk of recall bias, and because of the design of the study, the incidence of community-acquired pneumonia could not be established [21]. Our study extends current knowledge, as we were able to shed light on the incidence of pneumonia and influenza among professions with frequent person contacts, manual work and office work.

It has been identified that living with >10 people and living with children are factors associated with an increased risk of community-acquired pneumonia [10,11]. Further, a case-control study from Germany also identified that living with three or more children is associated with an increased risk of serologically confirmed influenza [12]. Our results are in line with these findings. We identified that people working in day care, nursing home care and public transportation were at an increased risk of pneumonia compared to people working within public administration. These professions are typically associated with direct person-to-person contact and include work with children. However, we found no increased rate of hospitalisation with pneumonia or influenza among people working within public schools, which may be explained by variations within the job description for this profession category.

An occupational study from England and Wales identified that welders and men who worked with exposure to metal fume or heated metal had a high mortality from pneumonia in the period from 1959 to 1990 [22]. Our study with contemporary data identified no increased associated risk of pneumonia in people working within the metal industry compared to people working within public administration. However, our study did not examine mortality or cause of death.

We identified a low rate of pneumonia and influenza in people within farming compared to people working within public administration. A study of crop farm workers identified an increased proportionate mortality ratio of several respiratory diseases compared to the non-agricultural population [23]. The discrepancy between studies may be explained by differences in the outcome studied.

We found that work within public transportation was associated with an increased rate of being hospitalised with influenza compared to work within public administration. In line with our finding, a case-control study from England was conducted during the 2008–2009 influenza season, which identified that people seeing their general practitioner (GP) for acute respiratory infection had more frequently used a bus or tram five days prior to GP contact compared to controls [24]. Our findings of hospitalisation with influenza should be interpreted with caution, as our data may be under-powered.

Influenza vaccination is an important factor in assessing the risk of hospitalisation with influenza. A previous study from 21 states in the USA investigated the coverage of influenza vaccination for a variety of professions [25]. This study identified that the coverage was highest among health-care practitioners and technical occupations (62.3%), and that people working within transportation and material-moving occupations were among the professions with low coverage (23.9%) [25]. The US labour market is difficult to compare to the Danish market. However, the US results may indicate that health-care workers in general are more likely to receive the influenza vaccination.

Our study has some limitations. First, the categorisation of professions was based on a standardised administrative system where specific details on job description such as number of person contacts were not available. Second, the status of influenza and pneumococcal vaccination was not available. However, only around 70,000 influenza vaccines were registered in the 2017–2018 season for healthy people <65 years of age, and it is therefore unlikely that differences in vaccination coverage explain our findings [26]. In Denmark, the influenza vaccine is recommended for people >65 years of age, people with chronic diseases, pregnant women in their second or third trimesters, patients with severe disease, patients on social welfare pension and relatives of patients with immunosuppression [27]. It is mandatory to register any vaccine in a central register in Denmark. Third, our data only provide information on pneumonia and influenza hospitalisation, and the overall societal burden of these diseases was not assessed. Fourth, multivariable adjusted regression analysis was conducted in order to assess confounders. However, based on the observational design, no causal link can be made. Residual confounding may be present. For instance, it would have been of interest to identify differences in urban and suburban areas of living. Fifth, the type of profession was assessed from the Danish Welfare Registry, which only includes people who have previously received financial support from the government. Although this includes the majority of the Danish population, it introduces an important selection in the cohort studied. Sixth, co-morbidity was assessed as diagnosis from an inpatient or outpatient visit at a hospital. However, data from general physicians were not included. This may have underestimated the true burden of co-morbidities.

In conclusion, we found that people working within public transportation, day care, nursing home care and sewers have a higher rate of being hospitalised with pneumonia compared to people working within public administration. People working in public transportation were at an increased risk of being hospitalised with influenza compared to people working in public administration. Our findings underline the need for investigations on ways to prevent pneumonia and influenza within the professions specified.

## Supplemental Material

SJP964974_Supplemental_Figures – Supplemental material for Work exposure and associated risk of hospitalisation with pneumonia and influenza: A nationwide study Click here for additional data file. Supplemental material, SJP964974_Supplemental_Figures for Work exposure and associated risk of hospitalisation with pneumonia and influenza: A nationwide study by Lauge Østergaard, Rikke Nørmark Mortensen, Kristian Kragholm, Michael Dalager-Pedersen, Kristoffer Koch, Lars Køber, Christian Torp-Pedersen and Emil Fosbøl in Scandinavian Journal of Public Health

SJP964974_Supplemental_Table – Supplemental material for Work exposure and associated risk of hospitalisation with pneumonia and influenza: A nationwide study Click here for additional data file. Supplemental material, SJP964974_Supplemental_Table for Work exposure and associated risk of hospitalisation with pneumonia and influenza: A nationwide study by Lauge Østergaard, Rikke Nørmark Mortensen, Kristian Kragholm, Michael Dalager-Pedersen, Kristoffer Koch, Lars Køber, Christian Torp-Pedersen and Emil Fosbøl in Scandinavian Journal of Public Health
