# Supplementary material for: Work exposure and associated risk of hospitalisation with pneumonia and influenza: A nationwide study
Source: Scand J Public Health. 2020 Oct 30;49(1):57–63. doi: 10.1177/1403494820964974 (PMC7859585; doi:10.1177/1403494820964974)
Supplement: SJP964974_Supplemental_Figures – Supplemental material for Work exposure and associated risk of hospitalisation with pneumonia and influenza: A nationwide study [file SJP964974_Supplemental_Figures.pdf]

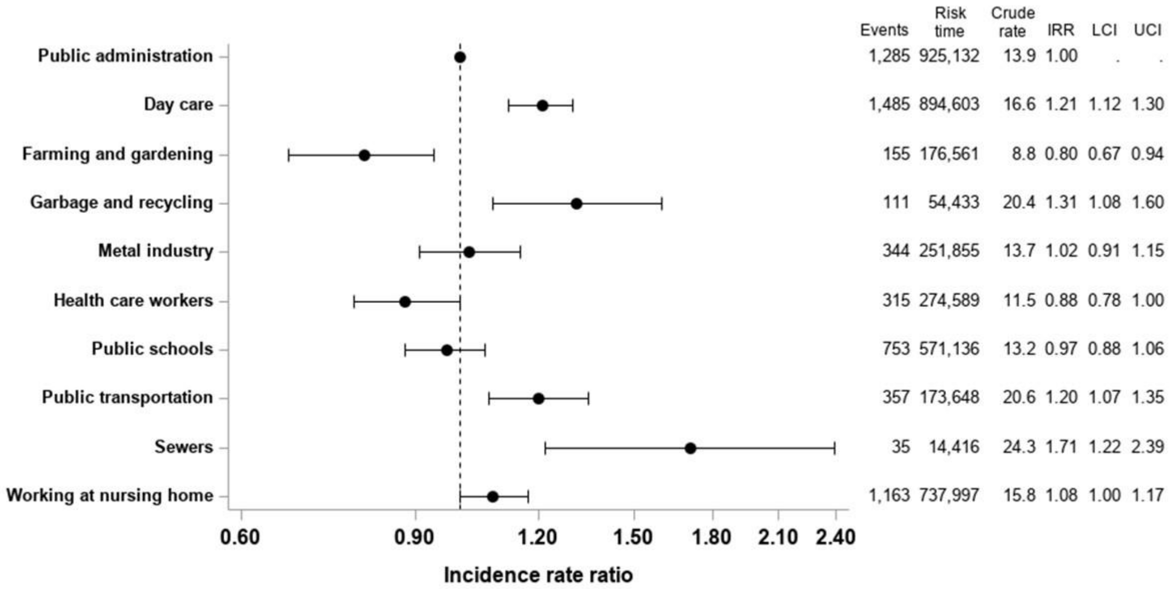

<u>Supplemental Figure 1. Risk of pneumonia by type of profession. Only primary diagnosis codes.</u>

Supplementary Figure 1. The figure shows the crude rate and incidence rate ratios of being hospitalized with pneumonia by type of profession including only primary diagnosis codes of pneumonia.

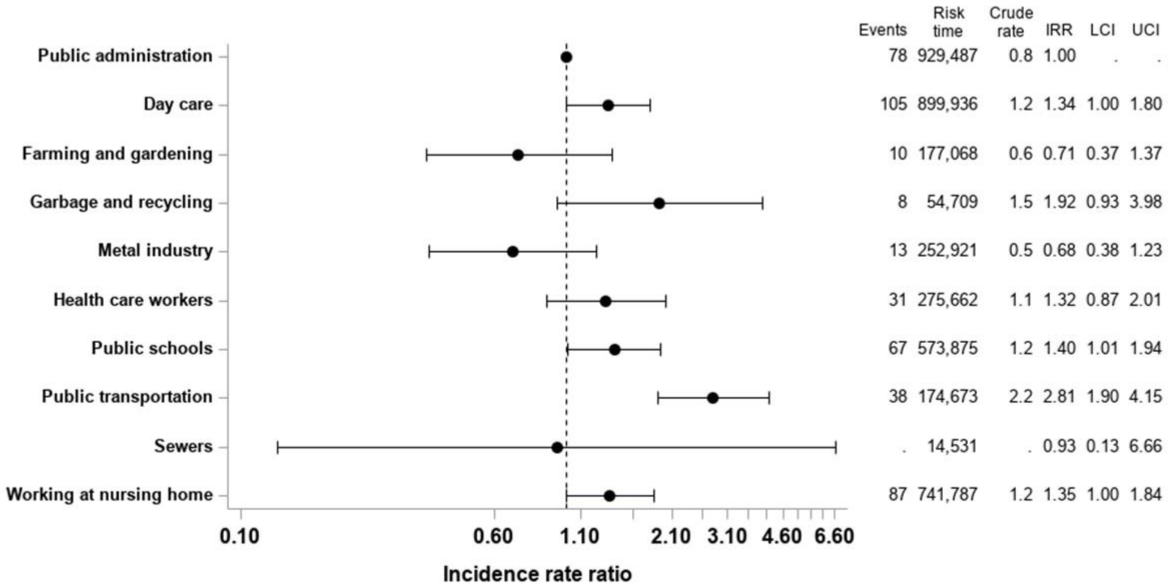

<u>Supplemental Figure 2. Risk of influenza by type of profession. Only primary diagnosis codes.</u>

Supplementary Figure 2. The figure shows the crude rate and incidence rate ratios of being hospitalized with influenza by type of profession including only primary diagnosis codes of influenza.

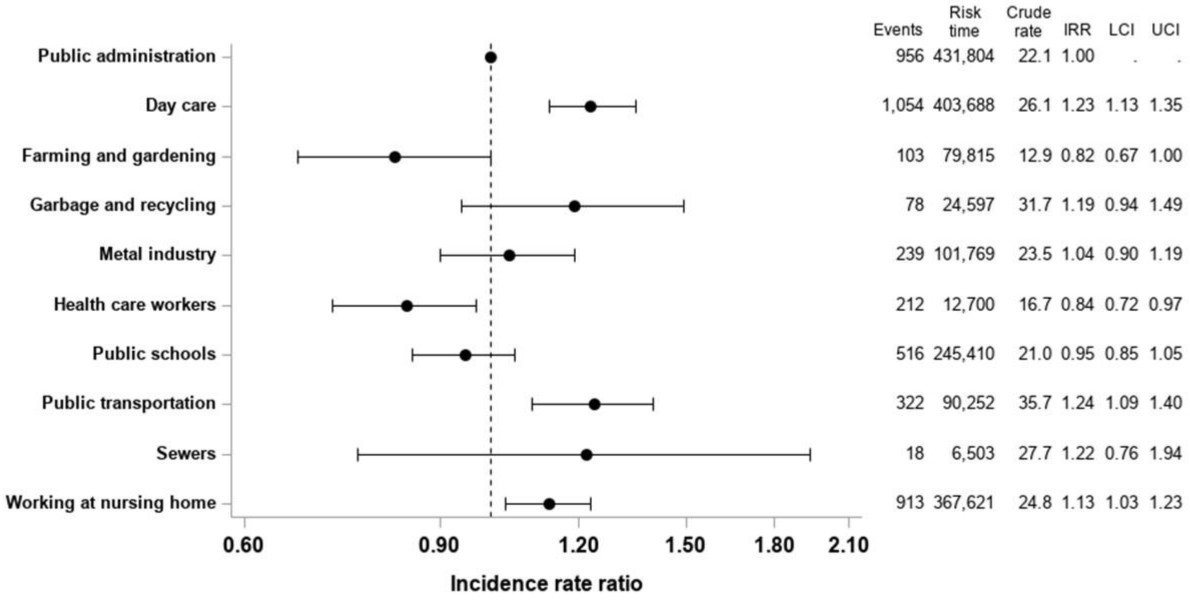

Supplemental Figure 3. Risk of pneumonia by type of profession. Persons >45 years.

Supplementary Figure 3. The figure shows the crude rate and incidence rate ratios of being hospitalized with pneumonia by type of profession including only persons over 45 years of age.

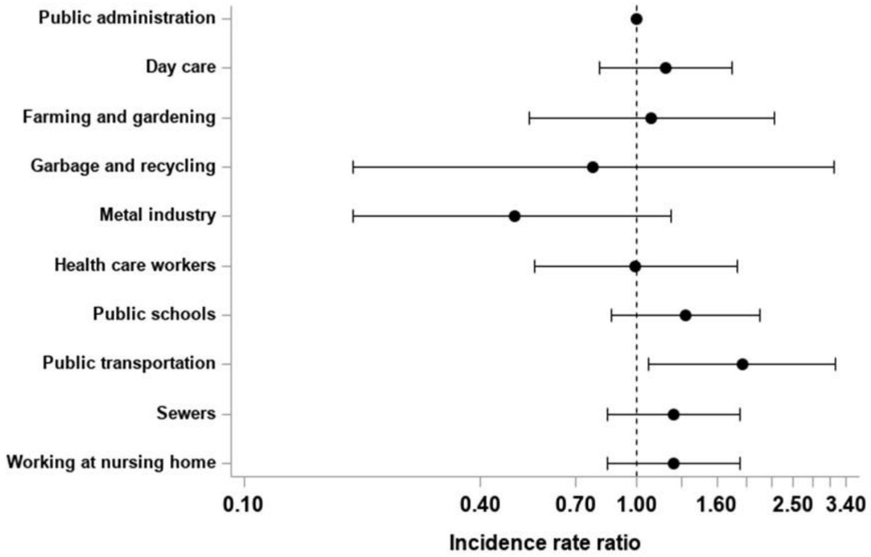

	Events	Risk time	Crude rate	IRR	LCI	UCI
Public administration	48	434,430	1.1	1.00	.	.
Day care	55	406,893	1.4	1.18	0.80	1.74
Farming and gardening	9	80,068	1.1	1.09	0.53	2.23
Garbage and recycling	.	24,798	.	0.77	0.19	3.17
Metal industry	5	102,465	0.5	0.49	0.19	1.22
Health care workers	14	127,629	1.1	0.99	0.55	1.80
Public schools	36	246,996	1.5	1.33	0.86	2.05
Public transportation	18	91,147	2.0	1.85	1.07	3.19
Sewers	.	6,575	.	1.24	0.84	1.83
Working at nursing home	53	370,285	1.4	1.24	0.84	1.83

Supplemental Figure 4. Risk of influenza by type of profession. Persons >45 years.

Supplementary Figure 4. The figure shows the crude rate and incidence rate ratios of being hospitalized with influenza by type of profession including only persons over 45 years of age.
