# Supplementary material for: Work exposure and associated risk of hospitalisation with pneumonia and influenza: A nationwide study
Source: Scand J Public Health. 2020 Oct 30;49(1):57–63. doi: 10.1177/1403494820964974 (PMC7859585; doi:10.1177/1403494820964974)
Supplement: SJP964974_Supplemental_Table – Supplemental material for Work exposure and associated risk of hospitalisation with pneumonia and influenza: A nationwide study [file SJP964974_Supplemental_Table.pdf]

Supplementary Table 1: Codes	
**Category**	**Codes**
Outcome	
Pneumonia	ICD-10: DJ12-18
Influenza	ICD-10: DJ09-11
Study population	
Farming and gardening	Danish profession code: 01
Metal industry	Danish profession code: 25
Sewers	Danish profession code: 37
Garbage and recycling	Danish profession code: 38
Public administration	Danish profession code: 84
Public transportation	Danish profession code: 49
Public schools	Danish profession code: 85
Health care workers	Danish profession code: 86
Nursing home care	Danish profession code: 87
Day care	Danish profession code: 88
Comorbidity	
Cancer	ICD10: DC00-DC97; ICD8: 140-209
Acute myocardial infarction	ICD-10: I21-24
Renal disease	ICD10: DN03-04, DN17-19, DR34, DI12-13; ICD8: 582-586, 588.
Chronic obstructive lung disease	ICD10: DJ42, DJ44; ICD8: 490-492
Heart failure	ICD10: DI42, DI50, DI110, DJ819; ICD8: 4270, 4271.
Acute myocardial infarction	ICD10: DI20-25; ICD8: 410-414.
Atrial flutter/fibrillation	ICD-10: DI48; ICD8: 4274.
Rheumatic disease	ICD-10: M05-06, M32-34, M353; ICD8: 7100, 7101, 7104, 7140, 7141, 7142, 7148, 725.
Peripheral vascular disease	ICD10: DI70, DI74; ICD8: 443.
Diabetes	ATC code: A10

ICD: international classification of diseases, ATC: Anatomical Therapeutical Classification System.
